# Ventriculo-atrial shunt in idiopathic intracranial hypertension

**DOI:** 10.1007/s00701-024-05985-4

**Published:** 2024-02-22

**Authors:** Sheikh M. B. Momin, Sophie R. Mullins, Claudia L. Craven, Laurence Watkins, Ahmed K. Toma

**Affiliations:** 1 Victor Horsley Department of Neurosurgery, National Hospital for Neurology and Neurosurgery, Queen Square, London, WC1N 3BG UK; 2 Institute of Inflammation and Ageing, University of Birmingham, Birmingham, B15 2TT UK; 3 Department of Neurosurgery, Addenbrooke’s Hospital, Cambridge, CB2 0QQ UK

**Keywords:** Idiopathic intracranial hypertension, Shunt, Ventriculo-atrial shunt, CSF, Hydrocephalus

## Abstract

**Purpose:**

CSF diversion  is a recognised intervention in idiopathic intracranial hypertension (IIH), particularly in the presence of vision-threatening papilledema. Although ventriculo-atrial (VA) shunt insertion is a routine neurosurgical procedure, ventriculoperitoneal and lumboperitoneal shunts have been mostly used in this particular indication. This study aims to look at a single centre’s experience with VA shunts in idiopathic intracranial hypertension (IIH).

**Methods:**

Retrospective case series with a review of electronic records over a 10-year period; exclusion criteria were duplication of same shunt insertion, no VA shunt insertion, paediatric patients and indication other than IIH. Notes were reviewed for demographics, shunt survival (defined by time prior to revision) and reasons for revision.

**Results:**

Eight VA shunt procedures were identified in 6 patients (mean age at insertion 34 ± 10 years) with a mean follow-up of 58 ± 25 months. All shunts were secondary procedures; 2 revisions from lumbo-pleural, 2 from ventriculopleural, 2 from ventriculoatrial and one each from ventriculoperitoneal and combined lumbo-/ventriculoperitoneal. At 50 months, 75% of VA shunts had survived, compared to only 58.3% of VPleural shunts in patients with IIH. Revisions were required due to acute intracranial bleed (1 case)—revised at day 1, and thrombus at distal site (1 case)—revised at day 57. Both shunts were later reinserted. From the latest clinic letters, all patients had their treatment optimised with this procedure, although only two patients had documented resolved papilloedema post-procedure.

**Conclusions:**

Ventriculo-atrial shunts are a safe and efficacious alternative option for CSF diversion in IIH. In this series, only 1 shunt was revised for a VA shunt-specific complication.

## Introduction

Idiopathic intracranial hypertension (IIH) is characterised by the presence of raised intracranial pressure (ICP) in the absence of hydrocephalus or a space-occupying lesion with a normal cerebrospinal fluid (CSF) composition [1, 2]. It typically affects obese women of childbearing age [3–5]. Symptoms can be debilitating and include headaches, pulsatile tinnitus, and visual disturbances—most commonly transient visual obscurations [6]. Additionally, it carries a risk of potentially irreversible visual loss [7, 8]. When sight is threatened, current UK guidelines recommend the use of CSF diversion or optic nerve sheath fenestration [9].

Unfortunately, due to the frequent comorbidity with other headache disorders, CSF diversion does not always control headache symptoms [10–12]. National guidelines recommend the insertion of a ventriculoperitoneal (VP) shunt where CSF diversion is indicated for IIH [9]. CSF diversion shunts are considered efficacious when there is a differential pressure between the proximal and distal end; when associated with high body mass index, IIH causes raised intra-abdominal pressure, thus potentially reducing this pressure gradient. This may lead to a lack of effective CSF drainage in IIH patients undergoing VP shunt insertion. Moreover, complications (CSF pseudocyst formation, abscess, adhesions, peritonitis, perforation and distal tip migration) [13–15], contraindications such as raised intra-abdominal pressure states (e.g. pregnancy) and adhesions from previous surgery may require alternative distal sites of a shunt. The most common options include the pleural cavity in ventriculo-pleural (VPleural) shunts and the right atrium in ventriculo-atrial (VA) shunts.

### Gap of knowledge

We have previously reported outcomes from a series of 5 IIH patients undergoing 7 VPleural shunt insertions at our centre, with a complication rate of 28.6% and a median shunt survival of 71.7% at 7 months [16]. However, the efficacy and complication rates of VA shunts in IIH have not yet been explored in detail in the literature. Al-Schameri and colleagues reported a single-centre cohort study of 255 patients undergoing VA shunt, 8 of whom had IIH [17]. However, there was no further subgroup analysis on outcomes for these IIH patients. The right atrium may theoretically be a more optimal distal site in IIH given that the right atrial pressure is more predictably 2–6 mmHg, allowing maintenance of the pressure gradient. Hence, the present study reports a single centre’s experience with VA shunts in IIH, survival of shunts, complications and efficacy.

## Methods and materials

### Study design

A single-centre retrospective case series of adult patients with VA shunts inserted for IIH, identified from electronic records, over a 10-year time frame (6 August 2013 to 6 August 2023).

### Participants

Inclusion criteria were (1) patients aged over 18 years when the VA shunt was inserted, (2) with a diagnosis of IIH as an indication of CSF diversion. All patients with alternative indications for CSF diversion, and patients under 18, were excluded. Demographic and clinical data collected included age, sex, number with a primary VA shunt, number with a secondary VA shunt (and previous VP or VPleural shunts) and the indication for VA shunt insertion.

### Outcome

Outcome data collected included time to VA shunt revision, number of revisions, indication for revisions and need for shunt adjustment and clinical outcomes, including headache management and neuro-ophthalmology review.

### Ventriculo-atrial shunt placement and technique

Ventriculo-atrial shunt insertion is a joint neurosurgery and interventional neuroradiology procedure at our centre. Under a general anaesthetic, the patient is positioned supine with their head on a horseshoe headrest. The ventricular catheter is placed in an identical fashion to regular VP shunt insertion. Using intraoperative ultrasound, usually with the assistance of interventional radiology, the internal jugular vein is identified, punctured and dilated to feed a guidewire to the right atrium. A distal catheter is then passed over the wire using a Seldinger technique. Using contrast media and intraoperative fluoroscopy, the destination of the distal tubing is determined, ensuring it is sitting within the superior cavo-atrial junction. Subcutaneous closure is performed with 2–0 Vicryl Plus (fast-absorbing polyglactin 910, Ethicon, Johnson and Johnson Medical Ltd.). The skin is closed with staples. Post-operative skull and anteroposterior and lateral chest radiograph are obtained to confirm that the distal catheter is optimally placed along the superior cavo-atrial junction.

### Statistical analysis

Shunt failure (end of survival) was defined as the time point at which the shunt had to be revised or removed. Survival analysis was performed using the Kaplan–Meier curves and the log-rank (Cox-Mantel) test. All statistical tests were performed on GraphPad Prism 6.0c.

## Results

### Patient characteristics

A total of 8 VA shunts were inserted with the intention to manage IIH symptoms between August 2013 and April 2023. These 8 procedures were performed in 6 patients. All patients identified were female with a mean age of 34 (range 19–46). All had had one previous type of shunt prior to VA shunt insertion, with three patients trialling more than one type of shunt.

### Shunt survival

**Fig. 1:**
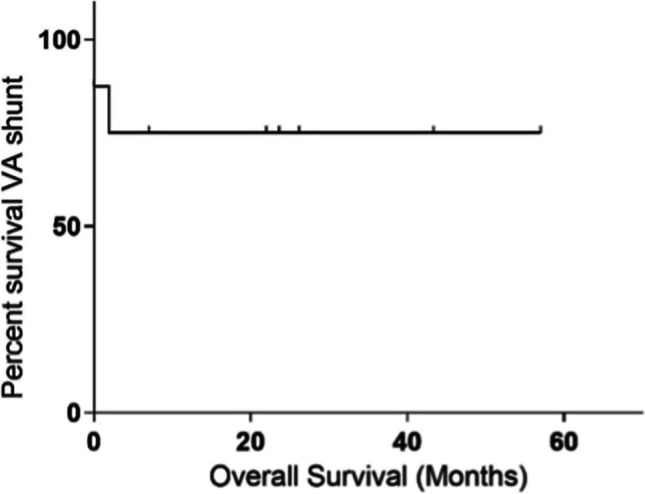
The Kaplan–Meier survival curve for VA shunts in our series (*n* = 8). Created with GraphPad Prism 6.0c

Of the 8 VA shunts inserted, one was removed on day 1 post-operatively due to an acute intracranial haemorrhage, later being re-inserted with no complications after 5 years and 8 months of follow-up at the time of the study. Moreover, one shunt was revised on day 57 due to a distal thrombus. At 50 months, 75% of VA shunts had survived (Fig. 1).

### Efficacy

Of the 6 patients, 3 markers were used to measure the efficacy of the shunts: ophthalmological assessment, symptom control and number of shunt adjustments.

Some ophthalmological assessments occurred in patients’ local hospitals; therefore, full data was not available. Two patients had documented the resolution of papilloedema. On follow-up, each patient had an improvement in headache, vision, or both symptoms. Moreover, each patient had at least 1 shunt adjustment.

## Discussion

### Study findings

VA shunting is known to be safe and as effective as other CSF shunting techniques for obstructive hydrocephalus and normal pressure hydrocephalus [17–20]. Hung and colleagues have even recommended VA shunting as a primary treatment for normal pressure hydrocephalus (NPH) [19].

However, there is little published data for the safety and efficacy of VA shunts specifically in the context of IIH. This study is the first to report on this cohort, and we demonstrate that VA shunts can be used as a valid second- or third-line treatment for IIH and indeed is quite effective at controlling symptoms. When compared to a series of patients undergoing VPleural shunt for IIH at our centre, 75% of VA shunts survived at 50 months, compared to 58.3% of VPleural shunts (*N* = 7) [16]. Whilst 2 shunts required removal, in both cases, they were later able to be replaced without subsequent complications on follow-up. It is of note that the acute intracranial bleed is a risk with all ventricular shunts regardless of distal site and therefore is not specific to VA shunts.

### VA shunts for symptom control in IIH

In this cohort, VA shunts provided good long-term control of symptoms of IIH, with two patients each having evidence of resolved papilloedema and superior control of headache symptoms.

The effective symptom control may owe to the relatively higher distal CSF pressures. Pressure in the right atrium is around 2–6 mmHg, higher than pressures within the peritoneal cavity (0–5 mmHg) and the pleural space (− 3.5 mmHg). It is possible that these higher pressures are more physiological for an IIH patient who, often for years, has tolerated raised ICP. Furthermore, a higher distal pressure may reduce over-drainage and siphoning, a problem that occurs in VPleural shunts [16].

### VA shunt safety in IIH

Despite our small cohort having a good safety profile, VA shunts are not without their well-reported and significant complications. Distal site-specific complications include obstruction, infection, thrombus formation, pulmonary emboli, pulmonary hypertension and shunt nephritis [21, 22]. There were no significant distal complications in our study, although this was a small cohort. The intracranial haemorrhage observed is not specific to VA shunts. The literature on outcome and safety profiles of VA and VPleural shunts in IIH patients is scarce, but six studies are presented in Table 1.

**Table 1:** A selection of articles from the literature on outcomes from VA/VPleural insertion for IIH, compared to the present study

Study	Primary pathology; (age group)	Number of shunts	Complication rate	Median shunt survival (range)	Revision rate	Symptom control
Current study	IIH adults; + VA shunt	8	28.6%	75% at 28 months (0.03–57 months)	28.6%	71.4%
Johnson et al. 1988 [23]	IIH adults; + VA shunt (-)	2	100%	–	100%	–
Sheth et al. 2009 [24]	IIH adults; + VA shunt (-)	–	No peri-procedural complications	–	–	–
Metellus et al. 2009 [25]	IIH adults; + VA shunts (-)	–	No peri-procedural complications	–	–	–
Craven et al. 2017 [16]	IIH adults; + VPleural shunt (-)	7	28.6%	71.4% at 7 months (1–33 months)	28.6%	71.4%

All studies on VA shunt insertion included IIH patients within the larger surgical cohort without further subgroup analysis, therefore precluding useful comparison to the present study. In Johnston et al.’s study, one patient underwent a secondary VA shunt insertion after persistent infection with valved lumboperitoneal and cisternoatrial shunts. However, this did not function satisfactorily and was removed after two revisions, being replaced with a cisternoatrial shunt [23]. Three studies described ultrasound-guided VA shunt insertion in a total of 11 patients [24–26], two of which described no peri-procedural complications [24–26]. Although complications arising from VA shunt insertion were described in these articles, this was not disaggregated to IIH patients’ outcomes. In Craven et al.’s study, 5 IIH patients had a VPleural shunt, with two revisions required due to unresolved symptoms [16]. The rate of shunt survival in this VPleural cohort is less than in our study with the VA cohort, but rates of complication, revision and symptom control are the same.

## Study strengths and limitations

Although this cohort is small, the findings are encouraging and conducting this study at a larger scale would be beneficial. The longest follow-up in this study was 6 years 8 months, which is long enough for late complications to manifest.

## Conclusion

Insertion of a VA shunt for IIH was efficacious for the management of our patients’ ICP, and symptoms were largely well controlled. One significant distal site-specific complication was noted; however, given the small cohort, the applicability of this to the IIH cohort at large is unclear. They remain a safe and effective second-line option where alternative distal sites have failed or are contraindicated and may also be considered first-line due to the reduced differential pressure gradient in the right atrium compared to the peritoneum in IIH.

## Data Availability

All data in this article is available on request.
